# Effect of hydrogen peroxide on the oxidative burst of neutrophils in pigs and ruminants

**DOI:** 10.14202/vetworld.2020.1934-1939

**Published:** 2020-09-22

**Authors:** Francesco Mosca, Abigail R. Trachtman, Jasmine Hattab, Giuseppe Marruchella, Pietro G. Tiscar

**Affiliations:** Department of Veterinary Medicine, University of Teramo, Loc. Piano d’Accio, 64100, Teramo, Italy

**Keywords:** hydrogen peroxide, luminol-derived chemiluminescence, pig, polymorphonuclear neutrophils, respiratory burst, ruminants

## Abstract

**Background and Aim:**

Neutrophils represent between 20% and 75% of white blood cells in animals and play a key role in an effective immune response. The generation of reactive oxygen species (ROS) is commonly referred to as an oxidative burst and is crucial under healthy and disease conditions. Interestingly, ROS are emerging as regulators of several neutrophil functions, including their oxidative burst. The present study aimed to investigate the effect of hydrogen peroxide on the oxidative burst of neutrophils, collected from domestic animal species (namely, pig, cattle, and sheep), and exposed to different stimuli.

**Materials and Methods:**

A total of 65 slaughtered animals were included in the present study: Twenty-two pigs, 21 cattle, and 22 sheep. Blood samples were collected at bleeding and neutrophils were then purified using *ad hoc* developed and species-specific protocols. Neutrophils were treated with hydrogen peroxide at micromolar-to-millimolar concentrations, alone, or combined with other stimuli (i.e., opsonized yeasts, and phorbol 12-myristate 13-acetate). The generation of ROS was evaluated using a luminol-derived chemiluminescence (CL) assay. For each animal species, data were aggregated and reported as mean area under curve±standard deviation. Finally, data were statistically analyzed by one-way ANOVA, followed by Tukey’s *post hoc* test.

**Results:**

Exposure of bovine and ovine neutrophils to hydrogen peroxide alone resulted in a dose-dependent enhancement of the CL response, which was significantly stronger at its highest concentration and proved particularly prominent in sheep. Opsonized yeasts and phorbol 12-myristate 13-acetate both proved capable of stimulating the generation of ROS in all animal species under study. Hydrogen peroxide negatively modulated the oxidative burst of neutrophils after exposure to those stimuli, observed response patterns varying between pigs and ruminants. Porcine neutrophils, pre-exposed to micromolar concentrations of hydrogen peroxide, showed a decreased CL response only to opsonized yeasts. Conversely, pre-exposure to hydrogen peroxide reduced the CL response of ruminant neutrophils both to yeasts and phorbol 12-myristate 13-acetate, the effect being most prominent at 1 mM concentration.

**Conclusion:**

These results indicate that hydrogen peroxide is capable of modulating the oxidative bursts of neutrophils in a species-specific and dose-dependent manner, substantial differences existing between pigs and ruminants. Further investigation is required to fully comprehend such modulation, which is crucial for the proper management of the generation of ROS under healthy and disease conditions.

## Introduction

Polymorphonuclear neutrophils (PMNs) (“neutrophils,” PMNs) are produced in the bone marrow and represent between 20% and 75% of blood circulating leukocytes in animals, with some relevant differences existing among species. In fact, higher percentage values are observed in most carnivore species (up to 75%), with intermediate values in the horse (about 50%) and pig (20-45%) and lower ones in domestic ruminants and laboratory rodents (20-30%) [1,2]. Under healthy conditions, the vast majority of PMNs remain quiescent and are disposed of, without ever being faced with any activating signals. On infection, PMNs are driven to infected tissues along the gradient of chemotactic molecules, which can be secreted by other cell types (“chemokines”) and/or provided by infectious agents. Therein, PMNs fight against bacteria and other microorganisms, playing a key role for an effective immune response [3-5].

The intra-phagosomal release of reactive oxygen species (ROS) is recognized as a crucial event in direct and/or indirect pathogen killing during phagocytosis. The generation of ROS is commonly referred to as an oxidative (“respiratory”) burst, a cellular process catalyzed by the multi-component and membrane-associated NADPH-oxidase complex. After exposure to inflammatory stimuli, NADPH oxidase assembles in the cell membrane and becomes active, thus generating superoxide anions (O_2_^-^), which spontaneously or enzymatically dismutate to hydrogen peroxide (H_2_O_2_). The latter is then converted into the bactericidal hypochlorous acid through the action of myeloperoxidase (MPO), an abundant enzyme contained in PMNs’ azurophil granules, and released into phagosomes and the extracellular microenvironment [1,3].

It is well known that ROS production can be modulated by a wide range of factors. Chemical mediators of inflammation (e.g., leukotriene B4), pro-inflammatory cytokines (e.g., tumor necrosis factor-alpha), and Toll-like receptor agonists (e.g., bacterial lipopolysaccharide) can prime PMNs, that is, can induce an intermediate state of activation, which boosts ROS generation, enhancing microbicidal activity. As with most biological processes, priming is reversible and finely regulated. Uncontrolled priming and/or impaired de-priming of PMNs result in an aberrant production of ROS, which are released into the external surrounding, contributing to tissue injury [4,6,7]. Excessive ROS accumulation impairs integrity and function of macromolecules (lipid, protein, and DNA), taking part in the pathogenesis of a wide range of disease processes, from cellular aging to inflammatory disorders, and malignant transformation of cells [5,7].

It is worthy to note that the generation of ROS is currently emerging as a regulator of several PMN functions [8], including the formation of “neutrophil extracellular traps” [9] and PMN death [10]. Some evidence suggests that micromolar concentrations of H_2_O_2_, compatible with those generated by activated PMNs and physiologically present at the site of inflammation, could affect the oxidative burst of human PMNs [11,12]. More recently, *Mycoplasma mycoides* subsp. *mycoides* was shown to enhance the oxidative burst of bovine PMNs through glycerol metabolism, used by mycoplasmas to produce H_2_O_2_ [13].

The present study aimed to investigate H_2_O_2_’s effect on the oxidative burst of PMNs, collected from domestic animal species (pig, cattle, and sheep) and exposed to different stimuli. The respiratory burst was evaluated through a luminol-derived chemiluminescence (CL) assay, which is widely employed and considered indicative of both intracellular and extracellular generation of ROS aside from superoxide and H_2_O_2_ [14].

## Materials and Methods

### Ethical approval

All samples were collected from animals routinely slaughtered in an abattoir approved by the European Community (Centro Carni Val Tordino, CE IT 2425 M, Mosciano Sant’Angelo, Teramo, Italy). Slaughtering procedures were performed strictly respecting the European legislation about the protection of animals at the time of killing (European Community Council Regulation No 1099/2009).

### Study period and location

The present study was conducted between November 2018 and October 2019 at the Laboratory of Veterinary Microbiology and Immunology “Louis Pasteur”, Department of Veterinary Medicine, University of Teramo.

### Animals

A total of 65 clinically healthy, regularly slaughtered animals were included in the present study: (a) Landrace×Large White pigs (n=22), aged 9-10 months, and weighing 145-165 kg; (b) Jersey breed cattle (n=21) aged 1-2 years and weighing 350-450 kg; and (c) Appenninica breed sheep (n=22) aged 2 years and weighing 45-55 kg.

### Blood sample collection and neutrophil isolation

At bleeding, the blood samples were collected in EDTA-containing tubes, refrigerated at 4°C and referred to the laboratory within 15 min. Species-specific protocols were developed and performed to isolate PMNs, as briefly reported below:

- Porcine PMNs – blood samples were diluted with PBS (1:1, v/v), layered onto Histopaque^®^-1077 (Merck KGaA, Germany) and centrifuged at 400× *g* for 40 min. Thereafter, the pellet was resuspended and incubated for 20 min in a lysis buffer (155 mM NH_4_Cl, 10 mM NaHCO_3_, and 0.12 mM EDTA) to completely remove erythrocytes. After washing with PBS and further centrifugation at 400× *g* for 10 min, a pellet mainly containing PMNs was obtained.
- Bovine PMNs – the blood samples were centrifuged at 1000× *g* for 20 min. The plasma, buffy coat, and about 50% of the red cell layer were then discarded. The remaining packed cell volume was incubated for 20 min in a lysis buffer to completely remove erythrocytes. After washing with PBS and further centrifugation at 400× *g* for 10 min, a pellet mainly containing PMNs was obtained.
- Ovine PMNs – blood was centrifuged at 400× *g* for 20 min. The plasma and buffy coat were then discarded. The remaining packed cell volume was incubated for 20 min in a lysis buffer, to completely remove erythrocytes. After centrifugation at 400× *g* for 10 min, the pellet was layered onto Histopaque^®^-1077 (Merck KGaA, Germany) and further centrifuged at 400× *g* for 40 min. Finally, after discharging Histopaque^®^-1077 and washing with PBS, a pellet mainly containing PMNs was obtained.

The purification of PMNs was microscopically assessed (Hemacolor® rapid stain kit Merck KGaA Germany), while their viability and concentrations were measured automatically (Vi-Cell, Beckman Coulter, USA). Only cellular suspensions showing ≥90% purified and viable PMNs were used, after being adjusted to 5×10^6^ cells/ml using PBS supplemented with 0.9 mM CaCl_2_, 0.2 mM MgCl_2_, and 5 mM glucose (pH 7.2).

An additional blood sample was collected from each animal in a serum-separating tube.

### Assessment of ROS production after neutrophil exposure to hydrogen peroxide

Neutrophils obtained from each animal were aliquoted, placed in 96-well plates (1×10^6^ cells/well), with luminol being then added to each well (final concentration=1 mM, Merck KGaA, Germany). Thereafter, H_2_O_2_ (Merck KGaA, Germany) was diluted with PBS and added to PMNs to reach the following final concentrations: 1 mM, 100 μM, and 10 μM.

Immediately after the addition of H_2_O_2_, the luminol-derived CL was monitored by a multi-mode plate reader (Sinergy H1, Bio-Tek, USA) for 30 min at 37°C, with a 1 min time interval between consecutive readings of the same well. The CL response was calculated by integrating the area under curve (AUC). For each animal species, data were aggregated and reported as mean AUC±standard deviation (SD) for each concentration of H_2_O_2_.

Preliminarily, H_2_O_2_’s effect on PMN viability was evaluated. Wells containing PMNs not exposed to H_2_O_2_ and wells lacking PMNs served as reference values.

### Effect of hydrogen peroxide on neutrophils stimulated to respiratory burst

After adding luminol (final concentration=1 mM, Merck KGaA, Germany) and exposure to different H_2_O_2_ concentrations (1 mM, 100 μM, and 10 μM) for 30 min, the PMNs’ respiratory burst was stimulated with *Saccharomyces cerevisiae* (Baker’s yeasts, Merck KGaA, Germany) at a final concentration of 5×10^7^ cells/well. Before use, the yeast was opsonized by incubation with an “autologous” serum (i.e., serum derived from the same animal) at 37°C for 30 min, and then washed and resuspended with PBS. Alternatively, the PMNs’ respiratory burst was induced with phorbol 12-myristate 13-acetate (PMA, Merck KGaA, Germany) at a final concentration of 1 μg/ml. For each blood sample, unstimulated PMNs, previously exposed or not to H_2_O_2_, acted as controls.

After adding the stimulus (i.e., yeast or PMA), the CL response was monitored as described above for 90 min at 37°C. For each animal species, data were aggregated and reported as mean AUC±SD for each concentration of H_2_O_2_.

### Statistical analysis

Data were analyzed by one-way ANOVA followed by Tukey’s *post hoc* test. The level for accepted statistical significance was p<0.01.

## Results

### Assessment of ROS production after neutrophil exposure to hydrogen peroxide

**Figure-1:**
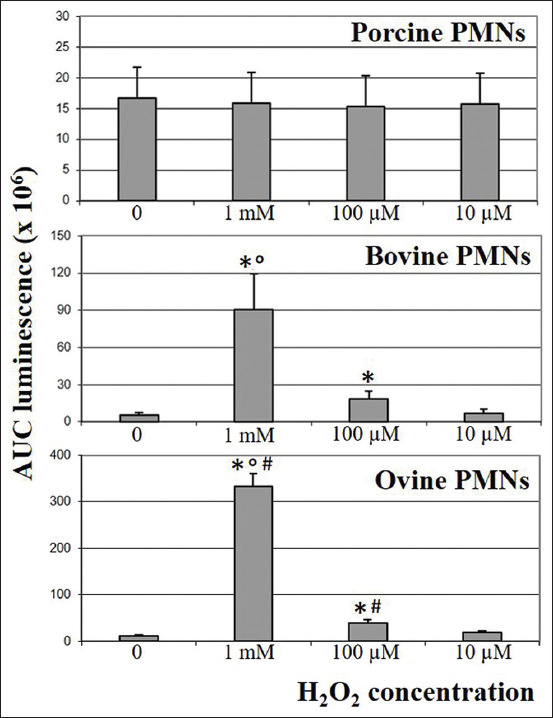
Chemiluminescence response after exposure to H_2_O_2_. Graphics show a clear-cut distinction between porcine and ruminant polymorphonuclear neutrophils (PMNs). Exposure to H_2_O_2_ had no significant effect on porcine PMNs (p>0.01). In contrast, the chemiluminescence (CL) response significantly increased after the exposure of bovine and ovine PMNs to 1 mM and 100 μM H_2_O_2_ (*p<0.01), when compared with controls (i.e., PMNs unexposed to H_2_O_2_, first column on the left side). In both ruminant species, the CL response was significantly higher at 1 mM concentration of H_2_O_2_ (p<0.01), when compared with 100 μM H_2_O_2_. In addition, it was significantly higher in sheep when compared with cattle (^#^p<0.01).

Data are graphically shown in Figure-1. Porcine PMNs demonstrated no significant change in terms of CL response after exposure to H_2_O_2_, regardless of its concentration. Conversely, bovine and ovine PMNs significantly increased their CL response when exposed to H_2_O_2_ at 1 mM and 100 μM concentrations. The CL response was particularly prominent at 1 mM concentration and significantly higher in sheep as compared with cattle.

Exposure to H_2_O_2_ did not affect the viability of PMNs, while no luminol-derived CL was detected in wells lacking PMNs.

### Effect of hydrogen peroxide on neutrophils stimulated to respiratory burst

Regardless of the animal species, PMNs unexposed to H_2_O_2_ showed a significant increase of luminol-derived CL after stimulation with opsonized yeasts or PMA, when compared with their respective negative controls (i.e., unstimulated PMNs).

#### Porcine neutrophils

**Figure-2:**
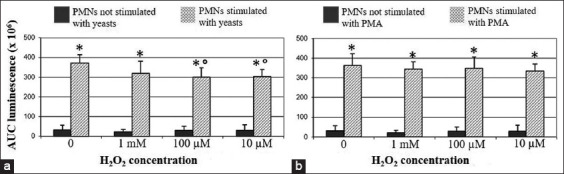
Porcine polymorphonuclear neutrophils (PMNs). Effect of H_2_O_2_ on the chemiluminescence (CL) responses after stimulation with yeasts or PMA. (a) Regardless of the exposure to H_2_O_2_, a strong and significant increase of the CL response (*p<0.01) was observed after stimulation with opsonized yeasts, when compared with their respective negative controls (i.e., PMNs not stimulated with yeasts). However, pre-exposure to 100 µM and 10 µM H_2_O_2_ slightly but significantly reduced the CL response after stimulation (p<0.01), when compared with PMNs not exposed to H_2_O_2_. (b) The stimulation with PMA induced a significant increase of CL response, when compared with negative controls (*p<0.01). Such CL response was not significantly changed by H_2_O_2_ at different concentrations.

Pre-exposure to H_2_O_2_ at final concentrations of 100 μM and 10 μM mildly but significantly reduced the CL response after stimulation with opsonized yeast, as compared with PMNs unexposed to H_2_O_2_. Conversely, H_2_O_2_ did not significantly affect the PMA-induced CL response, regardless of its concentration. All data regarding porcine PMNs are graphically shown in Figure-2.

#### Bovine neutrophils

**Figure-3:**
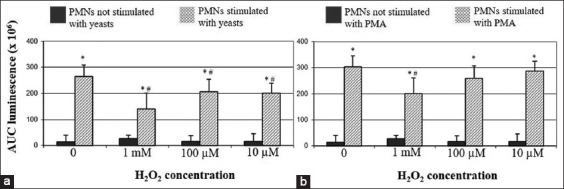
Bovine polymorphonuclear neutrophils (PMNs). Effect of H_2_O_2_ on the chemiluminescence (CL) responses after stimulation with yeasts or PMA. (a) Regardless of the exposure to H_2_O_2_, a strong and significant increase of the CL response (*p<0.01) was observed after stimulation with opsonized yeasts, when compared with their respective negative controls (i.e., PMNs not stimulated with yeasts). Pre-exposure to H_2_O_2_ significantly reduced the CL response after stimulation with yeasts (^#^p<0.01), when compared with PMNs not exposed to H_2_O_2_. (b) The stimulation with PMA always induced a significant increase of the CL response, when compared with negative controls (i.e., PMNs not stimulated with PMA; *p<0.01). However, such a CL response was significantly lower in PMNs pre-exposed to 1 mM H_2_O_2_ (^#^p<0.01), when compared with PMNs not exposed to H_2_O_2_.

Pre-exposure to H_2_O_2_ significantly reduced the CL response of bovine PMNs after stimulation with opsonized yeasts, when compared with PMNs unexposed to H_2_O_2_. The decreased CL response occurred regardless of H_2_O_2_ concentration and was particularly prominent at 1 mM concentration, the difference being statistically significant when compared with the 100 μM and 10 μM concentrations. The CL response to PMA appeared significantly lower only after pre-exposure to H_2_O_2_ at 1 mM concentration. All data regarding bovine PMNs are graphically shown in Figure-3.

#### Ovine neutrophils

**Figure-4:**
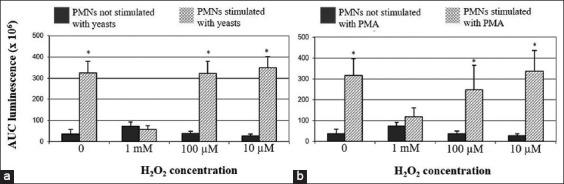
Ovine polymorphonuclear neutrophils (PMNs). Effect of H_2_O_2_ on the chemiluminescence (CL) responses after stimulation with yeasts or PMA. Pre-exposure to 1 mM concentration of H_2_O_2_ totally inhibited the CL response after stimulation with opsonized yeasts (a) or with PMA (b). Conversely, significant CL responses to yeasts or PMA were observed in PMNs not exposed to H_2_O_2_, or exposed to lower H_2_O_2_ concentrations (*p<0.01), when compared with unstimulated PMNs.

Pre-exposure to 1 mM H_2_O_2_ inhibited the CL response after stimulation both with opsonized yeasts and with PMA, no significant difference being evident when compared with unstimulated PMNs. Conversely, H_2_O_2_ did not significantly impair the CL response at the lower H_2_O_2_ concentrations. All data regarding ovine PMNs are graphically shown in Figure-4.

## Discussion

The oxidative burst represents a critical event both in healthy and disease conditions, playing a key role during the phagocytosis of microorganisms, as well as in the pathogenesis of a wide range of inflammatory-mediated disorders. It, therefore, appears ever more important to increase knowledge about the PMN oxidative burst. This is also particularly relevant from a therapeutic perspective, for the development of targeted anti-inflammatory drugs, which should reduce the generation of ROS without weakening the host defense against pathogen-derived stimuli [6,15,16].

Porcine PMNs showed no significant change of CL response after treatment with only H_2_O_2_, regardless of its concentration. Conversely, exposure of bovine and ovine PMNs to H_2_O_2_ resulted in a dose-dependent enhancement of the CL response, which was significantly stronger at the highest H_2_O_2_ concentration and proved particularly prominent in sheep. Overall, ruminant PMNs treated with only H_2_O_2_ behaved similarly to human neutrophils [11]. These findings raise a number of questions about the effects of “exogenous” H_2_O_2_ on PMNs’ functions, as well as about the molecular basis of such CL responses. According to Winn *et al*. [11], we may speculate at least the two following scenarios.

The H_2_O_2_ diffusion across cellular membranes occurs in a passive manner and is facilitated by specific aquaporin isoforms [17]. Therefore, as suggested for human PMNs [11], exogenous H_2_O_2_ could “freely” penetrate into azurophil granules and therein act as the substrate of MPO, enhancing the CL response. If so, differences among animal species might reasonably result from their varying ability to decompose H_2_O_2_, by means of intracellular enzymes [18]. Inter-species and age-related variations in activities of antioxidant enzymes have been reported in other tissue districts (namely, corneal epithelium) [19], but no comparative data are available about species-specific differences in antioxidant enzymes in PMNs.

More likely, H_2_O_2_ could injure cell membranes and promote PMN degranulation, thus allowing MPO to convert H_2_O_2_ to HOCl within the extracellular microenvironment [7,11]. Under this scenario, differences between pigs and ruminants might be due to the different susceptibility of their lipidic membranes to oxidative stress. In this respect, some data indicate that species differences exist in membrane susceptibility to lipid peroxidation [20,21], but further investigations are needed to support/rule out such a hypothesis.

We consider a more complete understanding of the biological significance of PMN’s response toward “physiologic” concentration of H_2_O_2_ to be particularly important. On infection, PMNs are the first cell type to arrive at the site of acute inflammation, where they recognize pathogens and are stimulated to respiratory burst. As a consequence, ROS (including H_2_O_2_) tend to accumulate outside the cells, where their enzymatic removal is less efficient [1,11,22]. Our data suggest that ruminant PMNs migrating later to the inflammatory site – when micromolar concentration of H_2_O_2_ is already present – could generate further H_2_O_2_, regardless of other stimuli. At least in cattle and sheep, as well as in human beings, such a self-perpetuating mechanism could create a microenvironment toxic to pathogens, thus having a defensive significance [11,22]. A similar defensive response could be also triggered by highly virulent mycoplasmas (e.g., African strains of *M. mycoides* subsp. *mycoides*), which produce H_2_O_2_ to damage host phagocytic cells [13,23]; paradoxically, host, and pathogen could use the same strategy for opposite purposes.

Opsonized yeasts and PMA are commonly used to trigger PMNs’ oxidative burst, acting through two distinct mechanisms; the former induces phagocytosis, while the latter activates the protein kinase C pathway [8]. According to the literature, the above stimuli both proved effective to stimulate ROS generation by PMNs from animal species under study [14,24-26].

Micromolar concentrations of H_2_O_2_ have been shown to suppress the luminol-derived CL response to surface-acting stimulants (i.e., zymosan and fMLP) by human PMNs, whereas it did not impair the response to PMA [11]. Accordingly, we observed that H_2_O_2_ negatively modulate the oxidative burst of PMNs collected from pigs, cattle, and sheep. However, once again we point out a different pattern of response between pigs and ruminants. After exposure to the lower H_2_O_2_ concentrations, porcine PMNs showed a decreased CL response to opsonized yeasts, while no effect was detected after stimulation with PMA. Conversely, pre-exposure to H_2_O_2_ reduced the CL response of ruminants’ PMNs both to yeasts and PMA, the effect being more prominent at 1 mM concentration. As previously suggested [11], the extracellular accumulation of H_2_O_2_ could moderate the oxidative burst acting as a negative feedback, especially in sheep and cattle. However, further knowledge is still needed in this field of research, aiming to properly manage such events.

## Conclusion

These results indicate that H_2_O_2_ is capable of modulating the oxidative bursts of PMNs in a species-specific and dose-dependent manner, substantial differences existing between pigs and domestic ruminants. Data obtained from pigs and ruminants partially match that reported in human beings. Further investigation is required for a complete comprehension of H_2_O_2_ modulation of the PMN oxidative burst. This is crucial to manage ROS generation under healthy and disease conditions.

## Authors’ Contributions

FM: Planning and supervision of the laboratory work, data analyses, writing of the manuscript. ART: Sample collection and laboratory work. JH: Sample collection and laboratory work. GM: Design of the work, data analyses and writing of the manuscript. PGT: Design of the work and data analyses. All authors read and approved the final manuscript.
